# Ultraviolet B Irradiation Reduces the Expression of Adiponectin in Ovarial Adipose Tissues through Endocrine Actions of Calcitonin Gene-Related Peptide-Induced Serum Amyloid A

**DOI:** 10.1371/journal.pone.0098040

**Published:** 2014-05-20

**Authors:** Sho Matsui, Takumi Yamane, Kazuo Kobayashi-Hattori, Yuichi Oishi

**Affiliations:** Department of Nutritional Science, Faculty of Applied Bioscience, Tokyo University of Agriculture, Tokyo, Japan; Albert Einstein College of Medicine, United States of America

## Abstract

Ultraviolet (UV) B irradiation decreases blood adiponectin levels, but the mechanism is not well understood. This study investigated how UVB irradiation reduces adiponectin expression in ovarial adipose tissues. Female Hos:HR-1 hairless mice were exposed to UVB (1.6 J/cm^2^) irradiation and were killed 24 h later. UVB irradiation decreased the adiponectin protein level in the serum and the adiponectin mRNA level in ovarial adipose tissues. UVB irradiation also decreased the mRNA levels of peroxisome proliferator-activated receptor (PPAR) γ, CCAAT/enhancer binding protein (C/EBP) α, C/EBPβ, and fatty acid binding protein 4 (aP2) in ovarial adipose tissues. In contrast, UVB irradiation increased the mRNA levels of interleukin (IL)-6 and monocyte chemoattractant protein (MCP)-1 in ovarial adipose tissues. In the serum and liver, the levels of serum amyloid A (SAA), involved in PPARγ, C/EBPα, C/EBPβ, aP2, IL-6, and MCP-1 regulation, increased after UVB irradiation. The *SAA* gene is regulated by IL-1β, IL-6, and tumor necrosis factor-α, but only IL-6 expression increased in the liver after UVB irradiation. Additionally, in the liver, hypothalamus, and epidermis, UVB irradiation increased the expression of calcitonin gene-related peptide (CGRP), which upregulates SAA in the liver. Collectively, our results suggest that the CGRP signal induced by skin exposure to UVB transfers to the liver, possibly through the brain, and increases SAA production via IL-6 in the liver. In turn, serum SAA acts in an endocrine manner to decreases the serum adiponectin level by downregulating factors that regulate adiponectin expression in adipose tissues.

## Introduction

Ultraviolet (UV) B irradiation is a major environmental factor that affects the structure and function of the skin. Transient strong UVB stimulation initiates an inflammatory response in the epidermis, resulting in roughening of the skin and barrier dysfunction. Recent studies showed that UVB irradiation affects not only the skin, but also the subcutaneous adipose tissues. Kim et al. reported that UVB irradiation of the skin reduced the expression of peroxisome proliferator-activated receptor (PPAR) γ and CCAAT/enhancer binding protein (C/EBP) α, which participate in adipocyte differentiation, in the subcutaneous adipose tissues [1]. Meeran et al. demonstrated that UVB irradiation reduced the plasma adiponectin level by affecting visceral adipose tissues [2]. Adiponectin, an adipokine secreted exclusively by differentiated adipose tissues, is mainly expressed in visceral adipose tissues. The expression of adiponectin is regulated by PPARγ, C/EBPα, C/EBPβ, fatty acid binding protein 4 (aP2), interleukin (IL)-6, and monocyte chemoattractant protein (MCP)-1. Adiponectin is abundant in the plasma of healthy humans (8.9±5.4 µg/mL) [3], whereas obese and overweight patients have low circulating levels of adiponectin. A low circulating level of adiponectin contributes to the pathogenesis of metabolic syndrome, acts as a risk factor for type 2 diabetes mellitus, and plays a role in the development of cardiovascular disease caused by abdominal obesity and insulin resistance [4], [5]. UVB irradiation decreases the level of plasma adiponectin [2], but the mechanism by which UVB irradiation decreases the expression of adiponectin in ovarial adipose tissues is still unclear.

UVB irradiation of the skin also increases the level of calcitonin gene-related peptide (CGRP) in the epidermis [6]. CGRP is a 37 -amino acid peptide produced by alternative splicing of the calcitonin-CGRP gene transcript. It is widely distributed in the central and peripheral nervous systems, skin, liver, heart, and blood vessels [7], [8]. Harada et al. indicated that locally expressed CGRP stimulated the nerves and promoted the synthesis and release of CGRP in several organs through the nervous system [9]. Furthermore, our previous study showed that CGRP treatment increased the expression of serum amyloid A (SAA), which is mainly regulated by IL-1β, IL-6, and tumor necrosis factor (TNF)-α in the liver. The increase in SAA might decrease the expression of adiponectin in adipose tissues [10]. Using DNA microarrays, we found that UVB irradiation increased CGRP expression in the epidermis, hypothalamus, and liver (data not shown). Therefore, we hypothesized that UVB-induced CGRP signals might transfer from the skin to other organs.

The purpose of this study was to examine how UVB-induced mediators decrease adiponectin mRNA levels in ovarial adipose tissues. We exposed the dorsal skin of hairless mice to UVB irradiation at 1.6 J/cm^2^ and measured adiponectin levels in the serum and adipose tissues. To investigate the mechanism by which UVB stimulation decreased the serum adiponectin level, we measured the mRNA levels of CGRP in the epidermis, hypothalamus, and liver; the mRNA levels of IL-1β, IL-6, TNFα, and SAA in the liver; and the mRNA levels of factors involved in adipocyte differentiation and hypertrophy in ovarial adipose tissues. Moreover, to clarify the endocrine action of the mediators, we determined the serum levels of CGRP, SAA, and IL-6.

## Materials and Methods

### Animals

Six-week-old female Hos:HR-1 mice (16–24 g; Japan SLC, Inc., Shizuoka, Japan) were housed individually in wire-bottomed cage under a 12 h light/dark cycle (lights on 8∶00.am) in a room maintained at 23–25°C with 50–56% relative humidity. Mice were given food and water *ad libitum*.

### Ethics statement

The animal protocol for this study was reviewed and approved by the Animal Care and Research Ethics Committee of Tokyo University of Agriculture (permission No. 090585).

### UVB irradiation

After one week of acclimatization, mice (n = 6) were exposed to a single dose of UVB irradiation (1.6 J/cm^2^) for 10 min using a UVB irradiation apparatus (TL20W-01; Orion Electric, Co., Ltd., Fukui, Japan) that delivers energy in the UVB (310–315 nm) wavelength range, with maximum energy at 311 nm. The mice were killed after exposure to UVB for 24 h. Another six mice, used as the non-UVB-irradiated control group, were exposed only to room light from the covered, non-UV emitting fluorescent tubes.

### Measurement of adiponectin, SAA, IL-6, and CGRP levels in the serum, hypothalamus, liver, and ovarial adipose tissues

Blood collected from the mice was centrifuged (3,000 rpm, 4°C, 20 min) to prepare serum samples. The hypothalamus and liver were homogenized using a Power Masher (Nippi Inc., Tokyo, Japan) with BioMasher (Nippi Inc.) in a lysis buffer containing 20 mM Tris (pH 7.4), 150 mM NaCl, 2 mM EDTA, 1% (v/v) Nonidet P-40, 0.1% (w/v) SDS, and protease inhibitor cocktail diluted 1∶100 (Sigma-Aldrich Inc., St. Luis, MO, USA). After centrifugation (12,000×g, 4°C, 10 min), the total protein in supernatant was measured using a reducing agent- and detergent-compatible (RC DC) protein assay reagent (Bio-Rad Laboratories Inc., Hercules, CA, USA) [11], and total protein concentrations of supernatant then diluted with lysis buffer to achieve 3 mg/mL concentrations. The level of adiponectin, SAA, IL-6, and CGRP proteins in all the samples were determined using the Mouse/Rat Adiponectin ELISA kit (Otsuka Pharm Co., Tokyo, Japan), PHASE™ RANGE Mouse SAA ELISA kit (Tri-Delta Diagnostics Inc., Morris Plains, NJ, USA), Mouse IL-6 ELISA Ready-SET-Go! kit (eBioscience Inc., San Diego, CA, USA), and CGRP ELISA kit (USCN Life Science Inc., Huston, TX, USA), respectively, according to the manufacturer's instructions.

### RNA extraction and quantitative polymerase chain reaction (PCR)

Fresh skin samples from mice were separated at the dermo-epidermal basement membrane as described by Trost et al. [12]. Total RNA was extracted from the epidermis, hypothalamus, liver, and ovarial adipose tissues using the SV Total RNA Isolation System (Promega Co., Madison, WI, USA). The samples were stored at −80°C until use. To synthesize cDNA, 2 µg of total RNA was reverse transcribed using a High Capacity cDNA Reverse Transcription kit (Applied Biosystems, Carlsbad, CA, USA), according to the manufacturer's instructions. The primers and probes for mouse *β-actin*, *adiponectin*, *PPARγ*, *C/EBPα*, *C/EBPβ*, *aP2, IL-6*, *MCP-1*, *SAA*, *IL-1β*, *TNFα*, and *CGRP* were obtained from Applied Biosystems as Assays-on-Demand primer/probe gene expression products. Real-time PCR was performed using the ABI PRISM 7300 Sequence Detection System with primers, probes, and the THUNDERBIRD™ Probe qPCR Mix (Toyobo Co., Osaka, Japan). PCR amplifications were performed in duplicate wells in the same 96-well plate under the following conditions: 2 min at 50°C, 10 min at 95°C followed by 50 cycles of 15 s at 95°C, and 1 min at 60°C. The mRNA levels of the targets were expressed relative to the level of mouse β-actin mRNA.

### Statistical analysis

The data were analyzed using Student's t-test in SPSS 21.0J (SPSS Inc., Chicago, IL, USA). The data were expressed as the mean ± standard error (SE). Results with *p*<0.05 were considered statistically significant. Pearson's product-moment correlation coefficient between the level of serum SAA and the mRNA level of adiponectin in ovarial adipose tissues or between the level of serum SAA and serum adiponectin in the UVB (−) and UVB (+) group was performed using SPSS 21.0J (SPSS Inc.).

## Results

### Effects of UVB irradiation on adiponectin levels in the serum and ovarial adipose tissues

**Figure 1:**
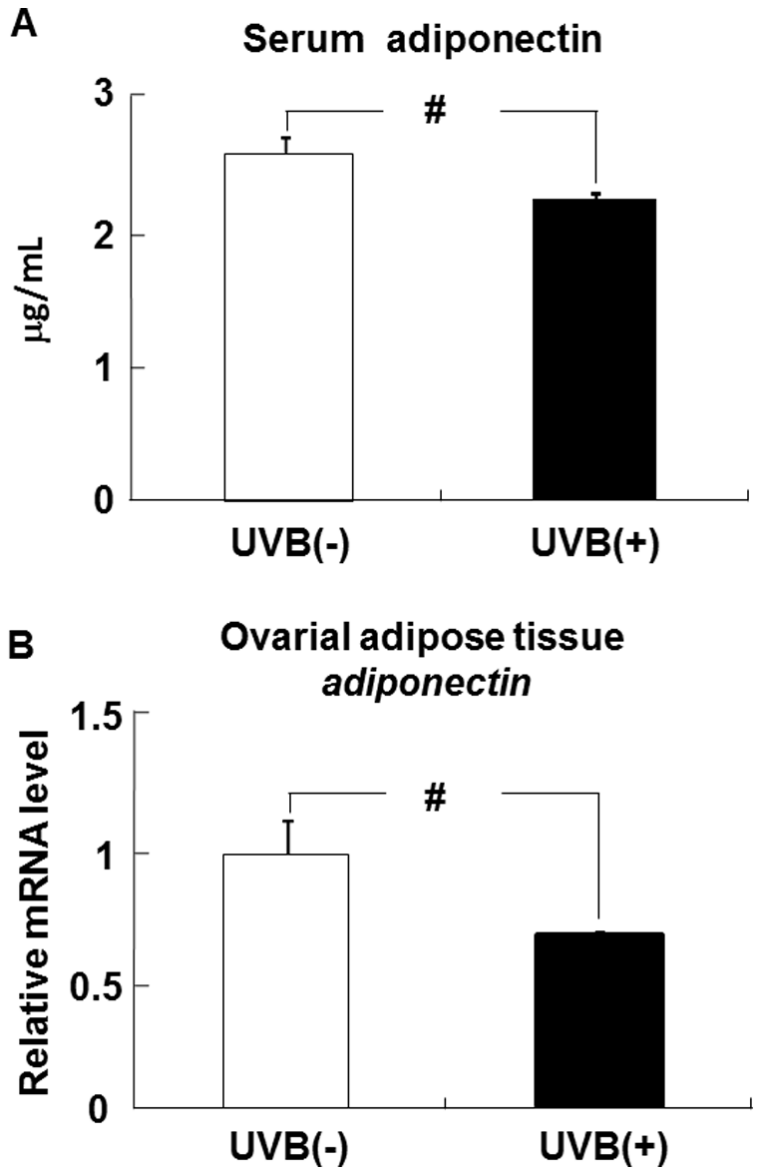
UVB irradiation decreases adiponectin levels in the serum and adipose tissues of hairless mice. The level of adiponectin in the serum (A) was measured by ELISA. The level of adiponectin mRNA in ovarial adipose tissues was determined by real-time PCR, and expressed relative to that of β-actin mRNA. Data represent the mean ±SE (n = 6). ^#^ *p*<0.05 indicates values that are significantly different from the UVB (−) group. UVB (−), non-UVB-irradiated mice; UVB (+), UVB-irradiated mice.

The serum level of adiponectin in the UVB irradiated (UVB (+)) group was significantly lower than that in the non-UVB irradiated (UVB (−)) group (Fig. 1A). In the ovarial adipose tissues, the adiponectin mRNA level in the UVB (+) group was significantly lower than that in the UVB (−) group (Fig. 1B).

### Effects of UVB irradiation on PPARγ, C/EBPα, C/EBPβ, aP2, IL-6, and MCP-1 mRNA levels in ovarial adipose tissues

**Figure 2:**
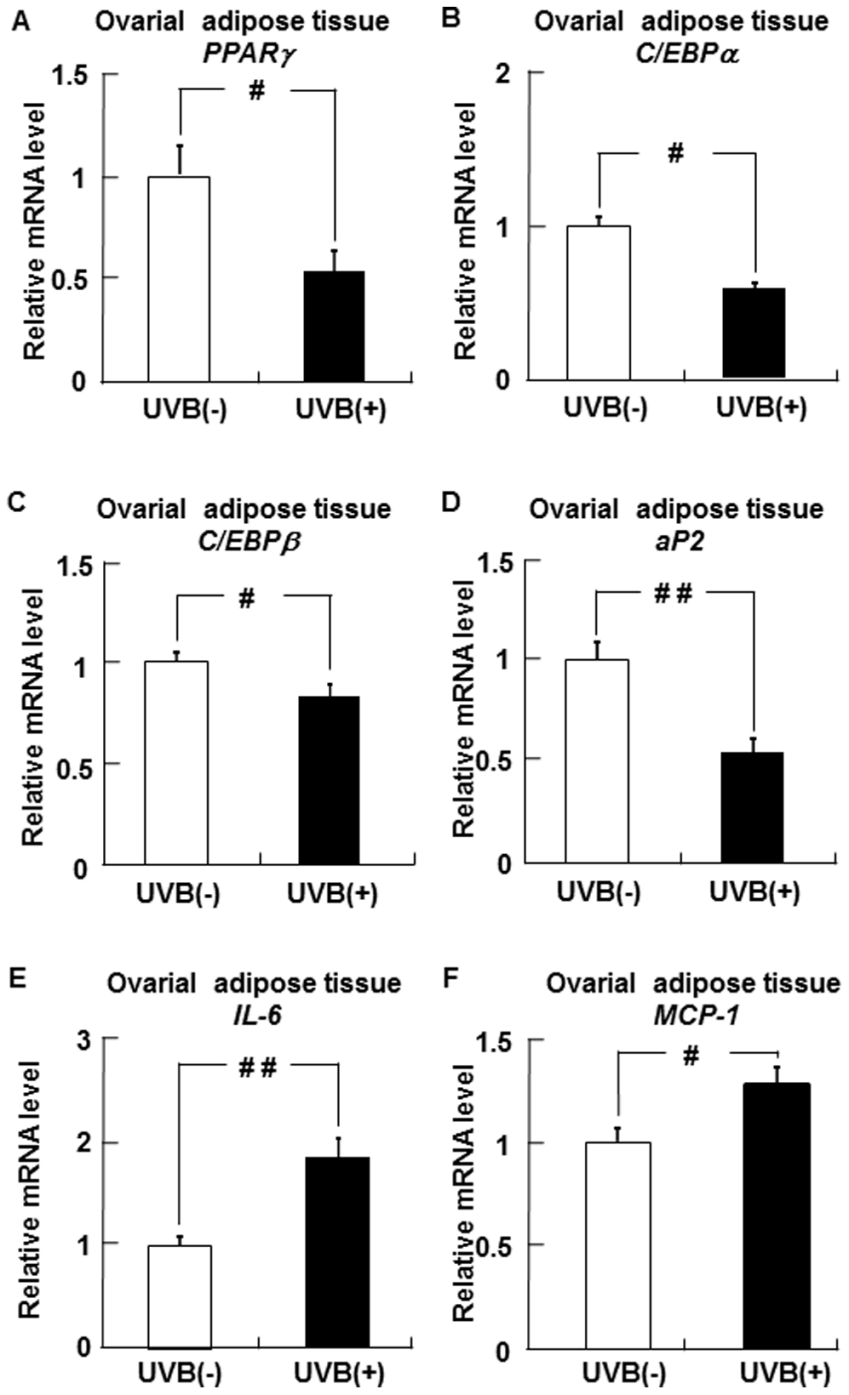
UVB irradiation PPARγ, C/EBPα, C/EBPβ, aP2, IL-6, and MCP-1 expression in hairless mice. PPARγ (A), C/EBPα (B), C/EBPβ (C), aP2 (D), IL-6 (E), and MCP-1 (E) mRNA levels in ovarial adipose tissues were measured by real-time PCR and expressed relative to the level of β-actin mRNA. Data represent the mean ±SE (n = 6). ^#^ *p*<0.05 or ^# #^ *p*<0.01 indicates values that are significantly different from the UVB (−) group. UVB (−), non-UVB-irradiated mice; UVB (+), UVB-irradiated mice.

Fig. 2A–D shows the mRNA levels of PPARγ, C/EBPα, C/EBPβ, and aP2, factors related to preadipocyte differentiation, in ovarial adipose tissues. PPARγ, C/EBPα, C/EBPβ, and aP2 mRNA levels in the UVB (+) group were lower than those in the UVB (−) group. On the other hand, the adipose mRNA levels of IL-6 and MCP-1, factors associated with adipocyte hypertrophy, were significantly higher in the UVB (+) group than in the UVB (−) groups (Fig. 2E and F).

### Effects of UVB irradiation on the serum SAA level

**Figure 3:**
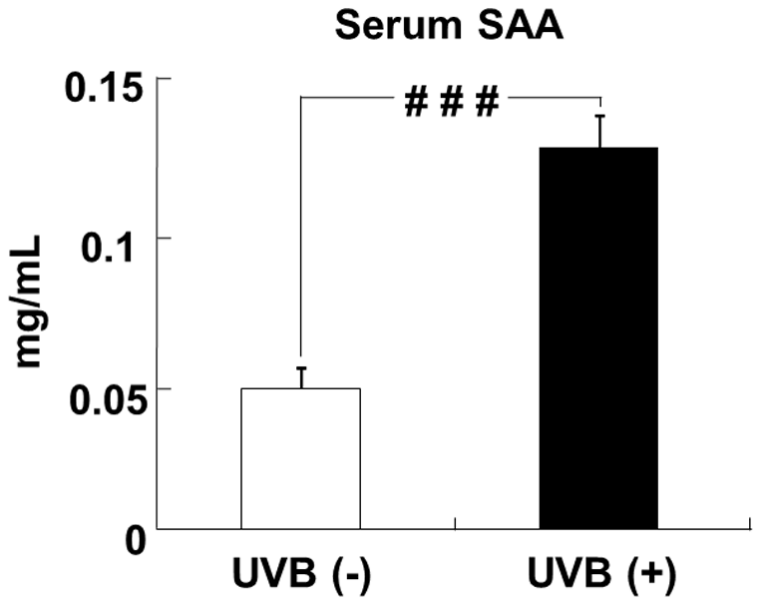
UVB irradiation increases the serum SAA level in hairless mice. The level of SAA in the serum was measured by ELISA. Data represent the mean ±SE (n = 6). ^# # #^ *p*<0.001 indicates values that are significantly different from the UVB (−) group. UVB (−), non-UVB-irradiated mice; UVB (+), UVB-irradiated mice.

The serum SAA protein level in the UVB (+) group was significantly higher than that in the UVB (−) group (Fig. 3).

### Correlation between SAA and adiponectin levels

**Figure 4:**
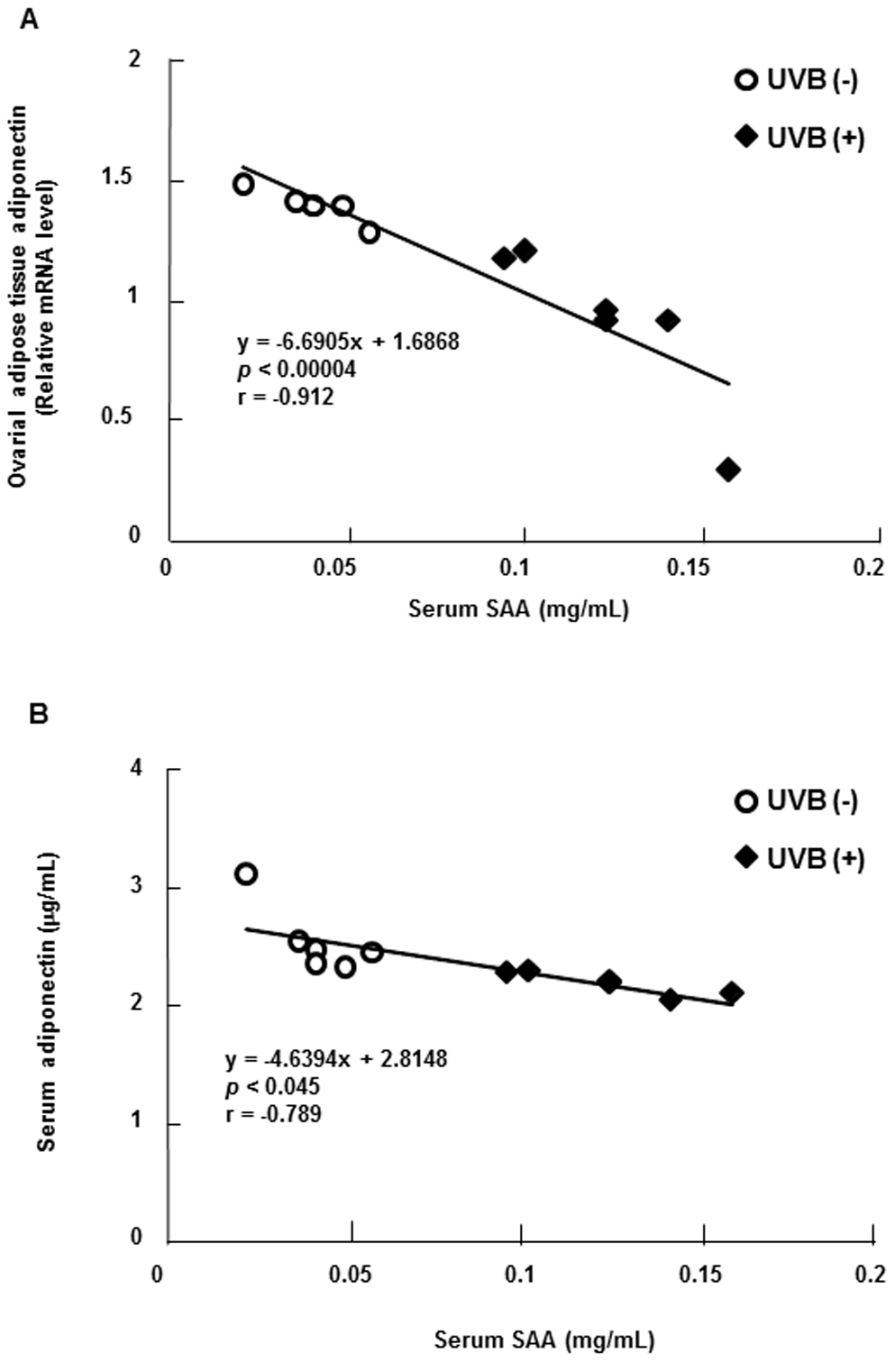
Correlation between SAA and adiponectin levels. SAA and adiponectin levels were measured as described in the Materials and Methods section. The correlation between the levels of SAA protein in the serum and adiponectin mRNA in ovarial adipose tissues or between the levels of SAA and adiponectin in the serum was evaluated by Pearson's product-moment correlation coefficient using individual data from 6 mice per UVB (−) and UVB (+) group.

The scatter plots in Fig. 4A and B revealed that serum level of SAA was negatively correlated with the mRNA level of adiponectin in ovarial adipose tissues (r = −0.912; p<0.00004; Fig. 4A) or the serum level of adiponectin (r = −0.789; p<0.045; Fig. 4B).

### Effects of UVB irradiation on SAA level in the liver

**Figure 5:**
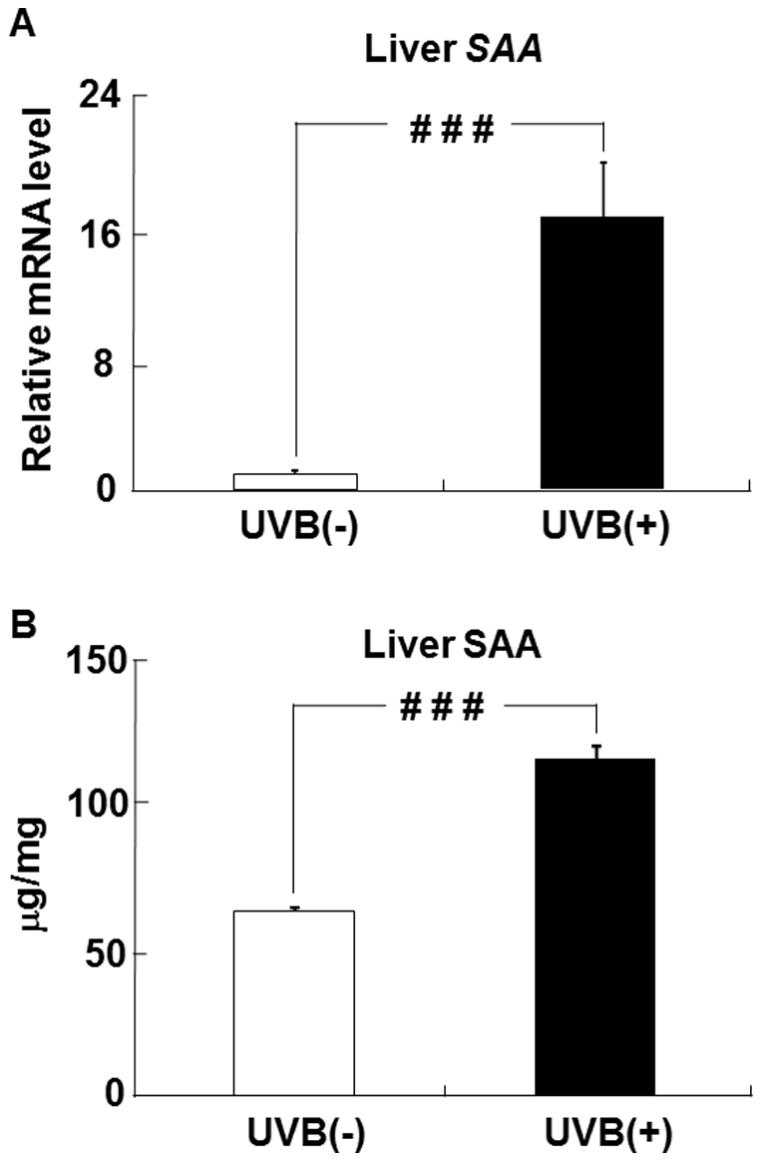
UVB irradiation increases liver SAA expression in hairless mice. The level of SAA mRNA in the liver (A) was measured by real-time PCR and expressed relative to that of β-actin mRNA. The level of SAA protein in the liver (B) was measured by ELISA. ^# # #^ *p*<0.001 indicates values that are significantly different from the UVB (−) group. UVB (−), non-UVB-irradiated mice; UVB (+), UVB-irradiated mice.

The level of hepatic SAA mRNA in the UVB (+) group was markedly higher than that in the UVB (−) group (Fig. 5A). The SAA protein level in the liver was also higher in the UVB (+) group than in the UVB (−) group (Fig. 5B).

### Effects of UVB irradiation on TNFα, IL-1β, and IL-6 levels in the liver

**Figure 6:**
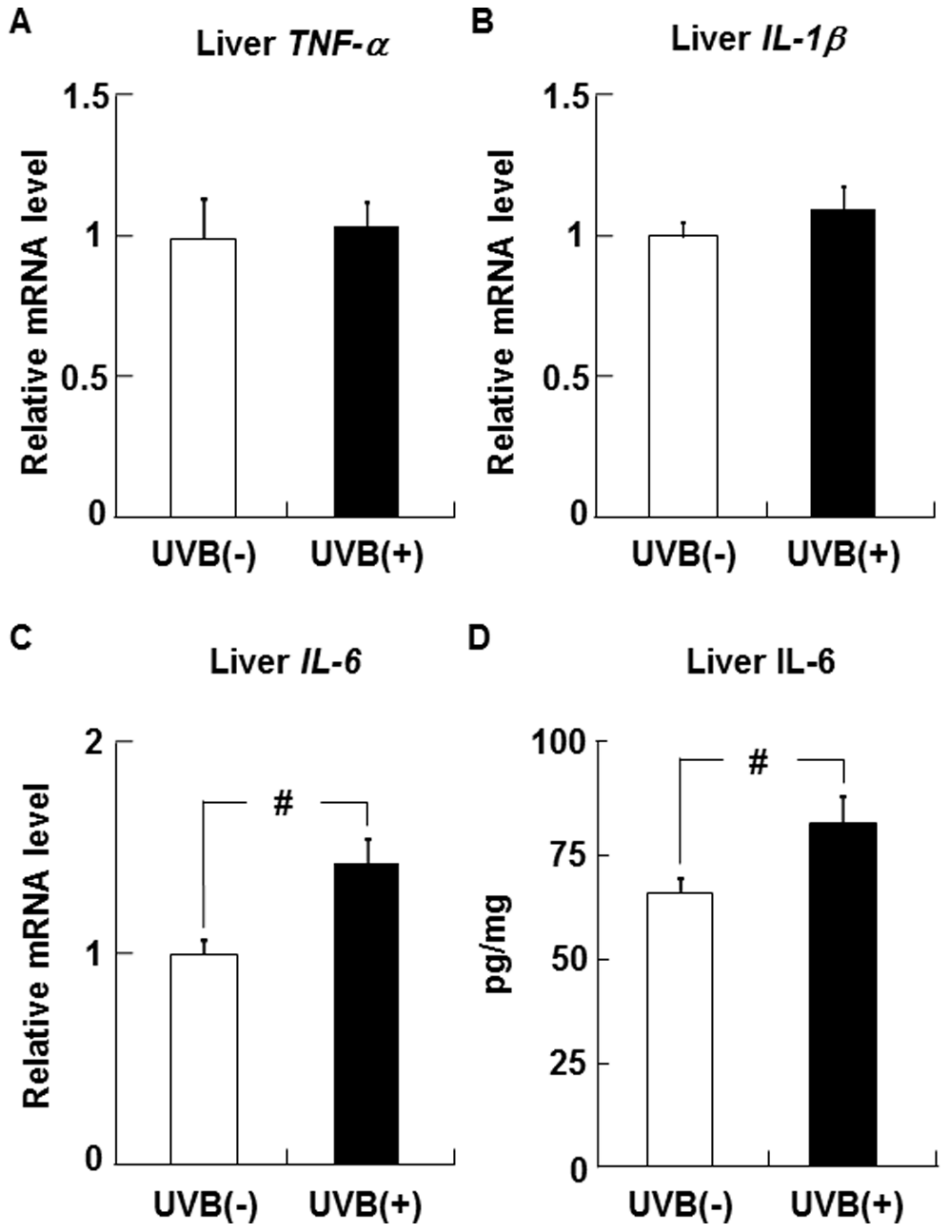
UVB irradiation on liver TNFα, IL-1β, and IL-6 expression in hairless mice. The levels of TNFα (A), IL-1β (B), and IL-6 (C) mRNA in the liver were determined by real-time PCR and expressed relative to the level of β-actin mRNA. The level of IL-6 protein in the liver was measured by ELISA (D). Data represent the mean ±SE (n = 6). ^#^ *p*<0.05 indicates values that are significantly different from the UVB (−) group. UVB (−), non-UVB-irradiated mice; UVB (+), UVB-irradiated mice.

No differences in the levels of TNFα and IL-1β mRNA in the liver were observed between the UVB (−) and UVB (+) groups (Fig. 6A and B). In contrast, hepatic IL-6 mRNA and protein levels were higher in the UVB (+) group than in the UVB (−) group (Fig. 6C and D).

### Effects of UVB irradiation on CGRP levels in the liver

**Figure 7:**
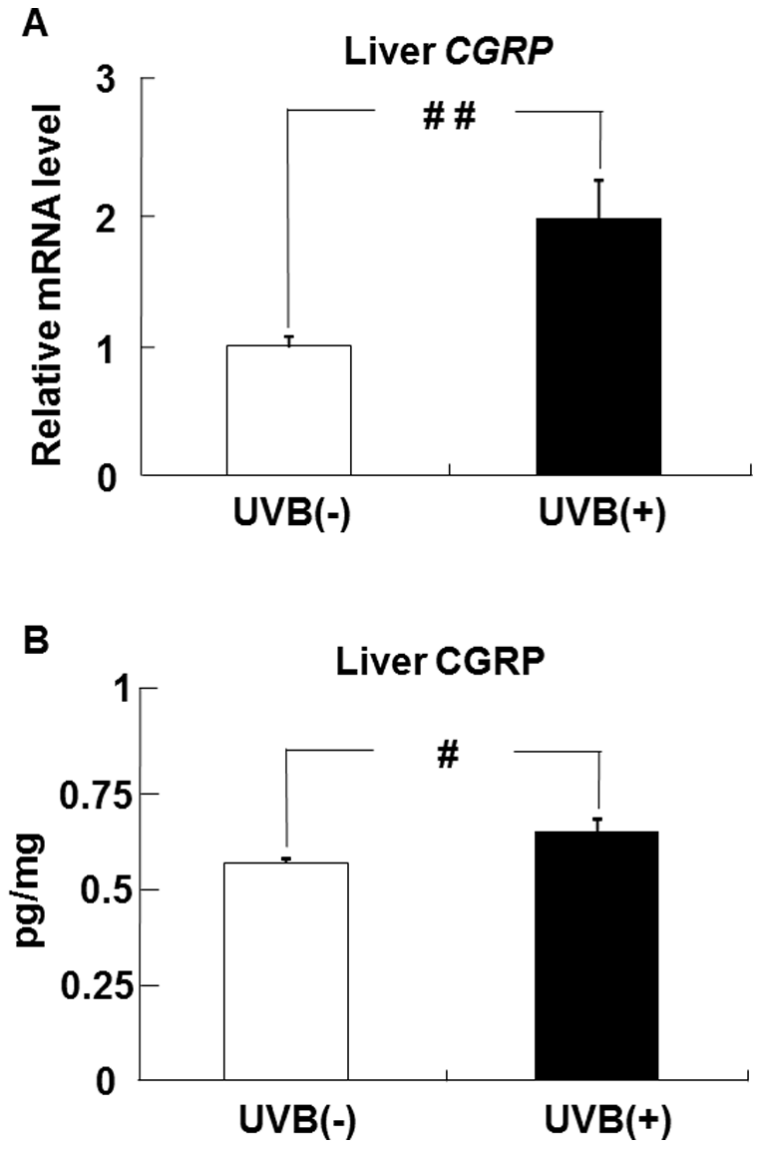
UVB irradiation increases CGRP expression in the liver of hairless mice. The level of CGRP mRNA in the liver (A) was measured by real-time PCR and expressed relative to that of β-actin mRNA. The level of CGRP protein in the liver (B) was measured by ELISA. ^#^ *p*<0.05 or ^##^ *p*<0.01 indicates values that are significantly different from the UVB (−) group. UVB (−), non-UVB-irradiated mice; UVB (+), UVB-irradiated mice.

The level of CGRP mRNA in the liver was higher in the UVB (+) group than in the UVB (−) group (Fig. 7A). The CGRP protein level in the UVB (+) group was also higher than that in the UVB (−) group (Fig. 7B).

### Effects of UVB irradiation on CGRP levels in the hypothalamus

**Figure 8:**
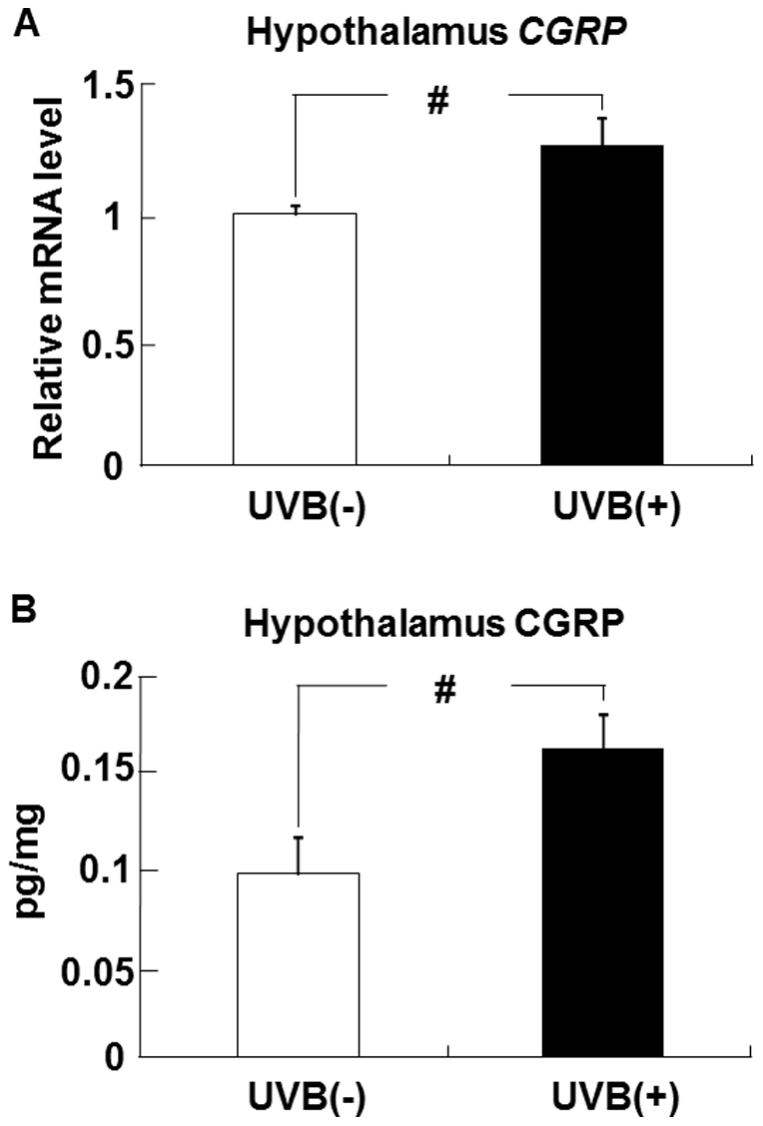
UVB irradiation increases CGRP expression in the hypothalamus of hairless mice. The level of CGRP mRNA in the hypothalamus (A) was determined by real-time PCR and expressed relative to that of β-actin mRNA. The level of CGRP protein in the hypothalamus (B) was measured by ELISA. Data represent the mean ±SE (n = 6). ^#^ *p*<0.05 indicates values that are significantly different from the UVB (−) group. UVB (−), non-UVB-irradiated mice; UVB (+), UVB-irradiated mice.

The level of hypothalamic CGRP mRNA in the UVB (+) group was higher than that in the UVB (−) group (Fig. 8A). Similarly, the CGRP protein level in the hypothalamus was higher in the UVB (+) group mice than in the UVB (−) group (Fig. 8B).

### Effects of UVB irradiation on CGRP levels in the epidermis, eye, and serum

**Figure 9:**
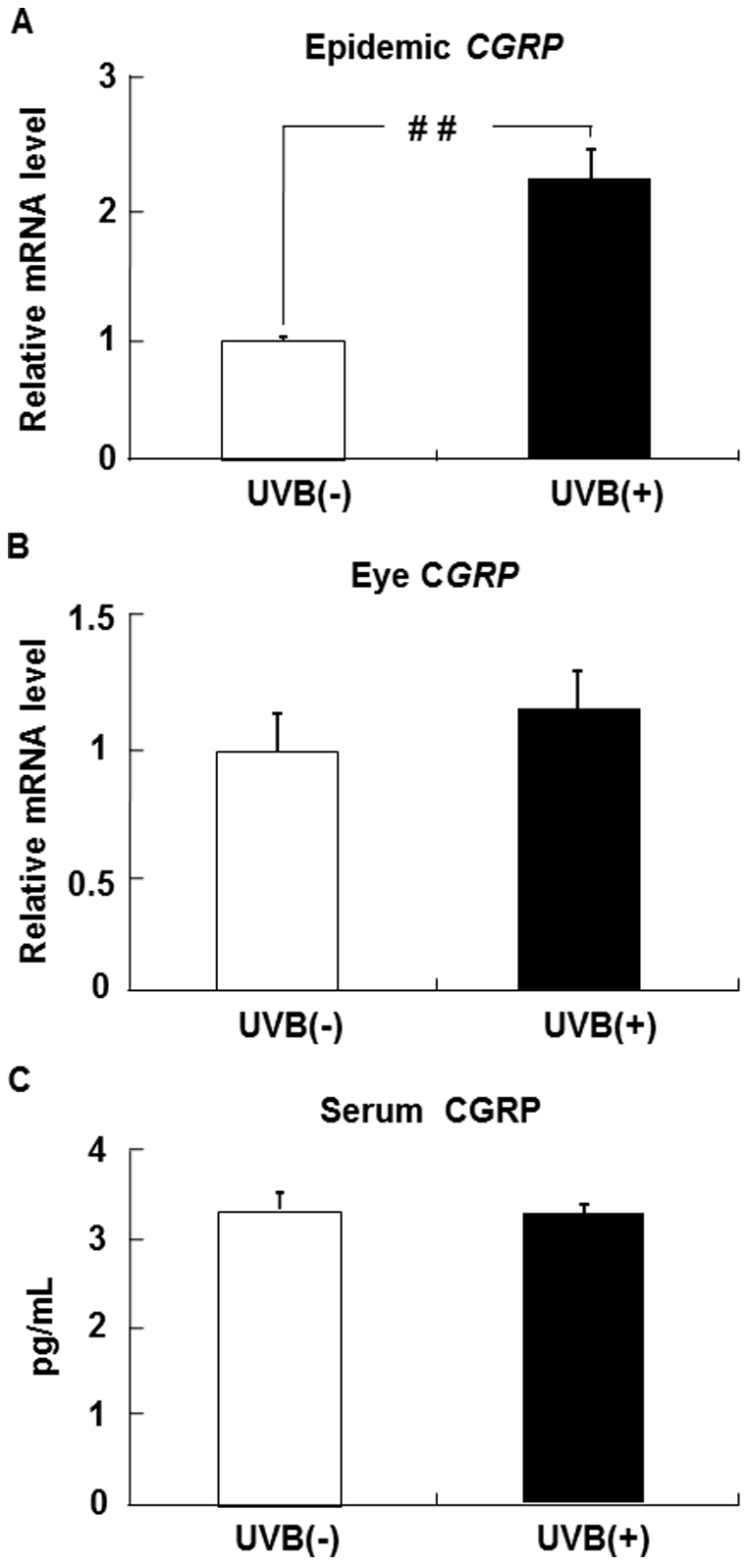
CGRP expression in the epidermis, eye, and serum of UVB-irradiated hairless mice. The levels of CGRP mRNA in the epidermis (A) and eye (B) were measured by real-time PCR and expressed relative to the level of β-actin mRNA. The level of CGRP in the serum (C) was measured by ELISA. Data represent the mean ±SE (n = 6). ^##^ *p*<0.01 indicates values that are significantly different from the UVB (−) group. UVB (−), non-UVB-irradiated mice; UVB (+), UVB-irradiated mice.

The epidermal CGRP mRNA level in the UVB (+) group was higher than that in the UVB (−) group (Fig. 9A). The CGRP mRNA level in the eye did not differ between the UVB (−) and UVB (+) groups (Fig. 9B). The level of CGRP protein in the serum was similar in the UVB (+) and UVB (−) groups (Fig. 9C).

## Discussion

Transient, strong UVB irradiation causes sunburn, tanning, wrinkling, aging, and cancer in the skin and cataracts in the eye [13], [14]. Because UVB irradiation does not penetrate far into the body, most of it is absorbed in the superficial tissue layers at a depth of 0.1 mm. Therefore, direct irritation from UVB is limited to the skin and eye, but signals induced by UVB irritation may be conveyed to the whole body after the primary reaction is initiated in the superficial layers. Indeed, UVB irradiation acts on ovarial adipose tissues and decreases the plasma adiponectin level [2]. However, the mechanism by which UVB irradiation decreases the blood adiponectin level is unclear. In the present study, to examine the mechanism underlying the decrease in the blood adiponectin level, we focused on mediators induced by UVB stimulation.

Adiponectin is synthesized and secreted exclusively by differentiated adipose tissues. In the present study, UVB irradiation significantly decreased the adiponectin levels in the serum and ovarial adipose tissues. The level of adiponectin in the blood is reduced in animal models of obesity [15], [16] and in human obesity, particularly in ovarial obesity [17]–[19]. Because adiponectin exerts a potent insulin-sensitizing effect by promoting the activation of AMP-activated protein kinase and PPARα in the liver and skeletal muscle, adiponectin may be a novel and promising therapeutic strategy for type 2 diabetes [20]. In addition, adiponectin is associated with alterations in skin biomechanical characteristics such as dermal layer thickness and elasticity [21], and with increases in collagen and hyaluronan production in dermal fibroblasts [22]–[24]. We previously demonstrated that reduction in the serum adiponectin level caused type I collagen and hyaluronan levels in the skin to decrease *in vivo*, which might lead to a decline in skin functions [25]. Therefore, direct damage to the skin by UVB irradiation as well as lower levels of serum adiponectin in response to UVB signals may cause skin functions to deteriorate.

To clarify the mechanism underlying the reduction in adiponectin mRNA levels in ovarial adipose tissues following UVB irradiation, we examined PPARγ, C/EBPα, C/EBPβ, aP2, IL-6, and MCP-1 mRNA levels in the adipose tissues. In adipocytes, increases in PPARγ, C/EBPα, C/EBPβ, and aP2 levels enhance adiponectin transcription, whereas increases in IL-6 and MCP-1 levels decrease adiponectin expression [26]–[31]. In our study, UVB irradiation decreased PPARγ, C/EBPα, C/EBPβ, and aP2 mRNA levels and increased IL-6 and MCP-1 mRNA levels in ovarial adipose tissues. These results suggest that the lower adiponectin level in ovarial adipose tissues after UVB irradiation results from the decreased expression of PPARγ, C/EBPα, C/EBPβ, and aP2, and the increased expression of IL-6 and MCP-1.

To identify protein that mediated the decrease in PPARγ, C/EBPα, C/EBPβ, and aP2 mRNA levels and the increase in IL-6 and MCP-1 mRNA levels, we measured the serum SAA level. SAA, which is mainly produced in the liver, is the earliest and most sensitive blood marker of acute inflammation [32], [33]. In the present study, UVB stimulation increased the serum SAA level. Moreover, Pearson's correlation test showed a negative correlation between the level of SAA in the serum and the level of adiponectin mRNA in ovarial adipose tissues and between SAA and adiponectin protein levels in the serum. In a time cours study of mice, Vieira et al. showed that the level of SAA in the serum positively correlated with the level of MCP-1 mRNA in ovarial adipose tissues and negatively correlated with the level of adiponectin in the serum [34]. A recent study using human multipotent adipose-derived stem cells showed that SAA decreased the expression of PPARγ, C/EBPα, C/EBPβ, and aP2 mRNA and increased the expression of IL-6 and MCP-1 mRNA [35]. Thus, our study suggests that SAA, induced by UVB, acts in an endocrine manner to reduce PPARγ, C/EBPα, C/EBPβ, and aP2 mRNA levels and increase IL-6 and MCP-1 mRNA levels in ovarial adipose tissues.

The secretion of pro-inflammatory cytokines such as IL-1β, IL-6, and TNFα in the liver regulates the expression of SAA [36]. Our results showed that UVB irradiation increased SAA, IL-6, and CGRP mRNA and protein levels in the liver but not change IL-1β and TNFα mRNA levels. Previously, we have shown that CGRP increased SAA mRNA and protein levels through the action of IL-6 in hepatocytes, but it did not affect IL-1β and TNFα mRNA levels [10]. Thus, the expression of SAA after UVB irradiation is probably upregulated by the CGRP-induced activation of IL-6. Given these results, it is highly likely that PPARγ, C/EBPα, C/EBPβ, aP2, IL-6, and MCP-1 gene expression in ovarial adipose tissues after UVB irradiation is regulated by SAA, which is induced by CGRP via IL-6.

**Figure 10:**
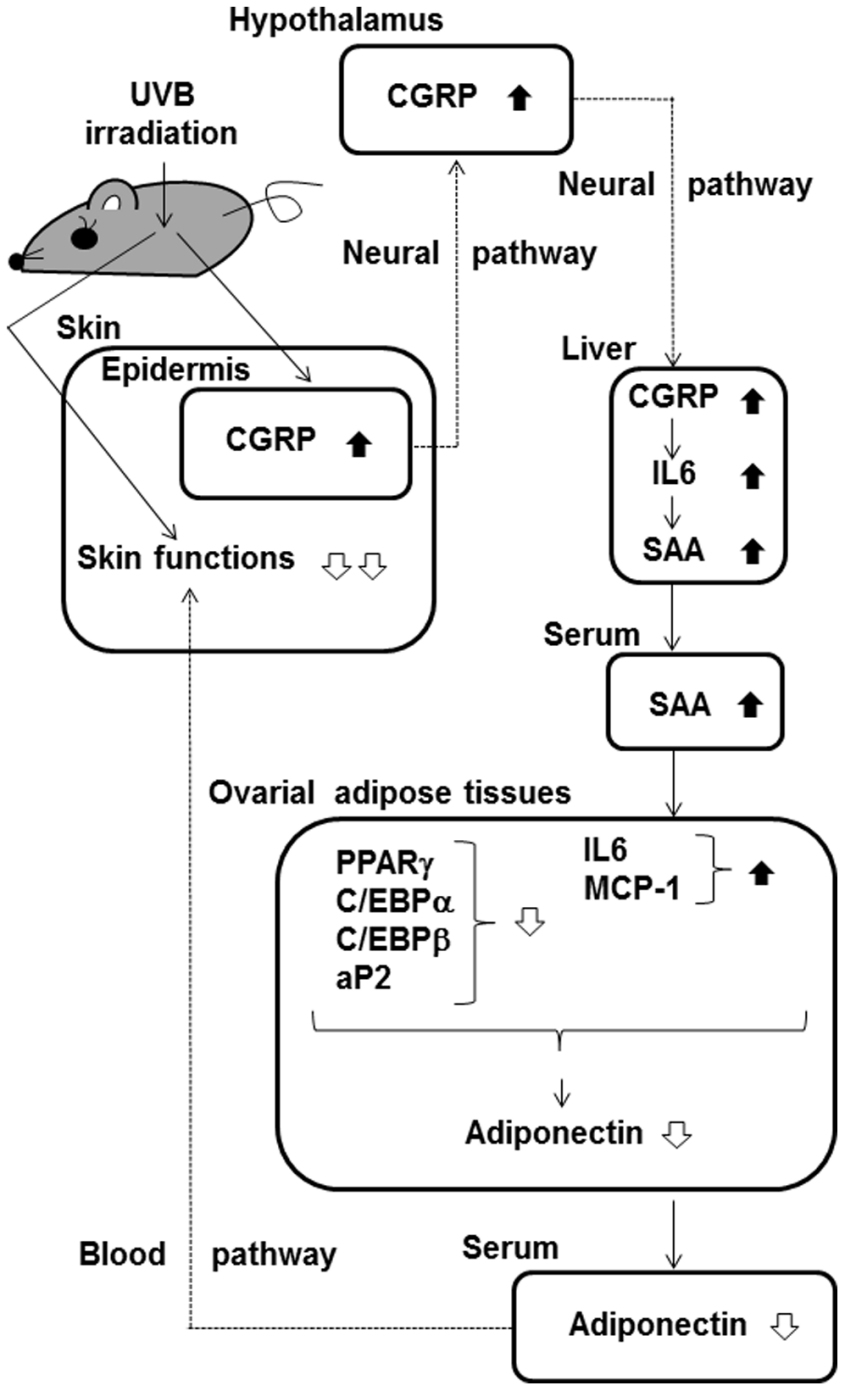
Putative mechanisms for the UVB-induced decrease in adiponectin expression in ovarial adipose tissues. The CGRP signal induced by exposure of the skin to UVB can transfer to the brain and then to the liver, possibly via a neural pathway. Increased CGRP in the liver induces the expression and secretion of SAA via the action of IL-6. In an endocrine manner, SAA in the serum downregulates PPARγ, C/EBPα, C/EBPβ, and aP2 mRNA levels and upregulates IL-6 and MCP-1 mRNA levels in the ovarial adipose tissues. The downregulation of adiponectin expression in the adipose tissues by these factors contributes to the decrease in serum adiponectin. Reduced levels of adiponectin in the serum may impair skin function (Yamane *et al*. 2010). Thus, the decrease in adiponectin induced by UVB irradiation can have adverse effects on skin function.

CGRP and its receptors are widely distributed in the nervous system and peripheral tissues, including the skin and liver [7], [8]. One of the roles of CGRP is inter-organ signal transduction. Harada et al. [9] indicated that sensory afferent information arising from the synthesis and release of CGRP was transmitted from the skin to other organs, leading to the synthesis and release of CGRP in other organs. Our study showed that UVB irradiation increased CGRP mRNA levels not only in the liver but also in the epidermis and hypothalamus. This finding suggests that UVB-induced CGRP signals transfer from the skin to the liver via the brain. Although CGRP and its receptors mediate the inflammatory response in the eye, such as that which occurs after UVB stimulation [37], [38], no change in the level of CGRP mRNA in the eye after UVB irradiation was observed in our study. This suggests that the decrease in adiponectin induced by UVB stimulation did not result from exposure through the eye. Our study also showed that UVB irradiation did not change the level of CGRP protein in the serum. This suggests that CGRP signals are not transduced via the blood. Putative mechanisms for the aforementioned effects, based on the results of this study and previous investigations, are shown in Fig. 10.

In conclusion, exposure of the skin to UVB decreased adiponectin levels in the serum and adipose tissues. The reduction in adiponectin was mediated by the endocrine action of SAA, which was induced by CGRP signals, possibly via the skin-brain-liver axis. Our study indicates that signals transfer from the skin to other organs after UVB irradiation, following an initial increase in epidermal CGRP. Our results may help elucidate the mechanisms by which UVB irradiation affects the whole body.
